# Effect of Poling on Photocatalysis, Piezocatalysis, and Photo–Piezo Catalysis Performance of BaBi_4_Ti_4_O_15_ Ceramics

**DOI:** 10.1002/gch2.202200142

**Published:** 2022-11-15

**Authors:** Pushpendra Kumar, Rahul Vaish, Tae Hyun Sung, Wonseop Hwang, Hyeong Kwang Benno Park, Anuruddh Kumar, Imen Kebaili, Imed Boukhris

**Affiliations:** ^1^ School of Engineering Indian Institute of Technology Mandi Mandi Himachal Pradesh 175005 India; ^2^ Department of Electrical Engineering Hanyang University 222, Wangsimni‐ro, Seongdong‐gu Seoul 04763 Korea; ^3^ Center for Creative Convergence Education Hanyang University 222, Wangsimni‐ro, Seongdong‐gu Seoul 04763 Korea; ^4^ Department of Physics Faculty of Science King Khalid University Abha 9004 Saudi Arabia; ^5^ Laboratoire de Physique Appliquée Groupe de Physique des matériaux luminescents Faculté des Sciences de Sfax Département de Physique BP 1171 Université de Sfax Sfax 3018 Tunisia; ^6^ Laboratoire des matériaux composites céramiques et polymères (LaMaCoP) Département de Physique Faculté des sciences de Sfax BP 805 Université de Sfax Sfax 3000 Tunisia

**Keywords:** BaBi _4_Ti _4_O _15_, methyl blue, photocatalysis, photo–piezocatalysis, piezocatalysis, poling, ultra‐violet radiation

## Abstract

This study focuses on analyzing the poling effect of BaBi_4_Ti_4_O_15_ (BBT) on the basis of photo and piezo‐catalysis performance. BBT powder is prepared via a solid state reaction followed by calcination at 950 °C for 4 h. BBT is characterized by an X‐ray diffractometer, scanning electron microscopy, Raman spectroscopy, and X‐ray photoelectron spectroscopy. The optical bandgap of BBT is evaluated with the help of Tauc's plot and found to be 3.29 eV, which comes in the photon energy range of ultra‐violet radiation. BBT powder is poled by using Corona poling in the presence of 2 kV mm^−1^ of electric field. An aqueous solution of methyl blue (MB) dye in the presence of UV radiation is used to evaluate the photo/piezocatalysis performance. Photocatalysis, piezocatalysis, and photo–piezo catalysis degradation efficiencies of poled and unpoled BBT powder are tested for 120 min of UV light irradiation. Photo–piezocatalysis shows degradation efficiencies of 62% and 40% for poled and unpoled BBT powder, respectively. Poling of BBT powder shows significant enhancement in degradation performance of MB dye in aqueous solution. Scavenger tests are also performed to identify reactive species.

## Graphical abstract

BaBi_4_Ti_4_O_15_ (BBT) powder is prepared by a solid state reaction with a calcination temperature of 950 °C for 4 h. Half of the BBT powder is poled by corona poling (electric field of 4 kV mm^−1^ for 4 h). Poled and unpoled BBT powder is tested for photo, piezo, photo–piezo catalysis with methylene blue dye. Results show that poling significantly increases the degradation efficiency.

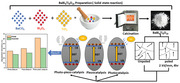

## 1 Introduction

Photocatalysis has an important technique for degrading organic contaminants in air and water. Different chemical redox reactions generated by photoexcited charge carriers in an excited semiconductor can effectively degrade dangerous organic contaminants from industrial wastewater.^[^ ^1^ ^]^ Traditional semiconductor photocatalysts, such as TiO_2_, CdS, ZnO, and WO_3_, have limited photocatalytic activity due to quick recombination of photo‐induced electrons and holes before they can commence photocatalytic activities, which is one of the most significant difficulties for commercial applications of these semiconductors.^[^ ^2^ ^]^ Ferroelectric materials as photocatalysts are considered to be a promising catalyst to overcome this problem in typical semiconductor photocatalysts.^[^ ^3^, ^4^ ^]^ Ferroelectrics’ polar structure creates an internal electric field, which aids in the passage of photoinduced charge carriers and so improves their separation. Typical ferroelectrics with perovskite structures, such as BaTiO_3_ and BiFeO_3_, have been shown to have photocatalytic activity.^[^ ^5^, ^6^, ^7^ ^]^ Furthermore, noble metals (such as Ag, Au, Pt, and others) can be added to perovskite materials to improve their plasmonic properties and construct effective photocatalysts.^[^ ^8^, ^9^, ^10^ ^]^ Despite the fact that significant progress has been achieved in studying the photocatalytic capabilities of ferroelectric materials with perovskite structures, it is critical to investigate alternative ferroelectric materials with other crystal structures for possible photocatalytic applications.

Intergrowth of [Bi_2_O_2_]^2+^ layers and pseudo‐perovskite blocks (A_n−1_B_n_O_3n+1_)_2_, *n* = number of perovskite‐like layers, produce the Aurivillius phases. Due to their remarkable qualities, they have gotten a lot of attention. Their high Curie temperature makes them appealing for high‐temperature piezoelectric devices.^[^ ^11^ ^]^ Their high polarizations and fatigue‐free abilities are the reason, so that they are also promising materials for non‐volatile ferroelectric random‐access memory applications.^[^ ^12^ ^]^ Apart from the application discussed above, researchers are making an effort to use these materials as photocatalysts. Kudo^[^ ^13^ ^]^ and Tang et al.^[^ ^14^ ^]^ were the first to report that Bi_2_WO_6_ has photocatalytic activity and could decompose organic molecules when exposed to visible light. Following that, further Aurivillius phase materials with photocatalytic behavior were discovered, notably Bi_3_TiNbO_9_,^[^ ^15^ ^]^ SrBi_2_Ta_2_O_9_,^[^ ^16^ ^]^ Bi_6_Ti_3_WO_18_,^[^ ^17^ ^]^ and Bi_4_Ti_3_O_12_.^[^ ^18^ ^]^ BaBi_4_Ti_4_O_15_ (BBT) is also an Aurivillius oxide with relaxor ferroelectric properties.^[^ ^19^ ^]^ The crystal structure and electrical properties of BBT single crystals or ceramics have been studied.^[^ ^20^, ^21^, ^22^, ^23^, ^24^, ^25^ ^]^ Wenzhi Qi et al. were the first to report photocatalysis of Rhodamine B (RhB) solution.^[^ ^26^ ^]^

While most of the literature focuses on BaTiO_3_, LiNbO_3_, and similar compounds for piezocatalysis, Aurivillius family materials are not exposed thoroughly. Hence, this article focuses on studying the effect of poling on photocatalytic activity of BBT. BBT was synthesized by solid state reaction. BBT was calcinated at 950 °C for 4 h. After synthesis of BBT, it is characterized by X‐ray diffractometer (XRD), scanning electron microscopy (SEM), Raman spectroscopy and X‐ray photoelectron spectroscopy (XPS). Catalytic performance is evaluated by methyl blue (MB) aqueous in deionized water. Catalytic behavior is evaluated for photo/piezo/photo–piezocatalysis. Band gap of BBT was evaluated with the help of Tauc's plot and found to be 3.29 eV. Degradation efficiencies were evaluated for photo, piezo, photo–piezo catalysis with poled and unpoled BBT as catalyst.

## 2 Results and Discussions

### 2.1 Characterization of Catalyst

**Figure 1:**
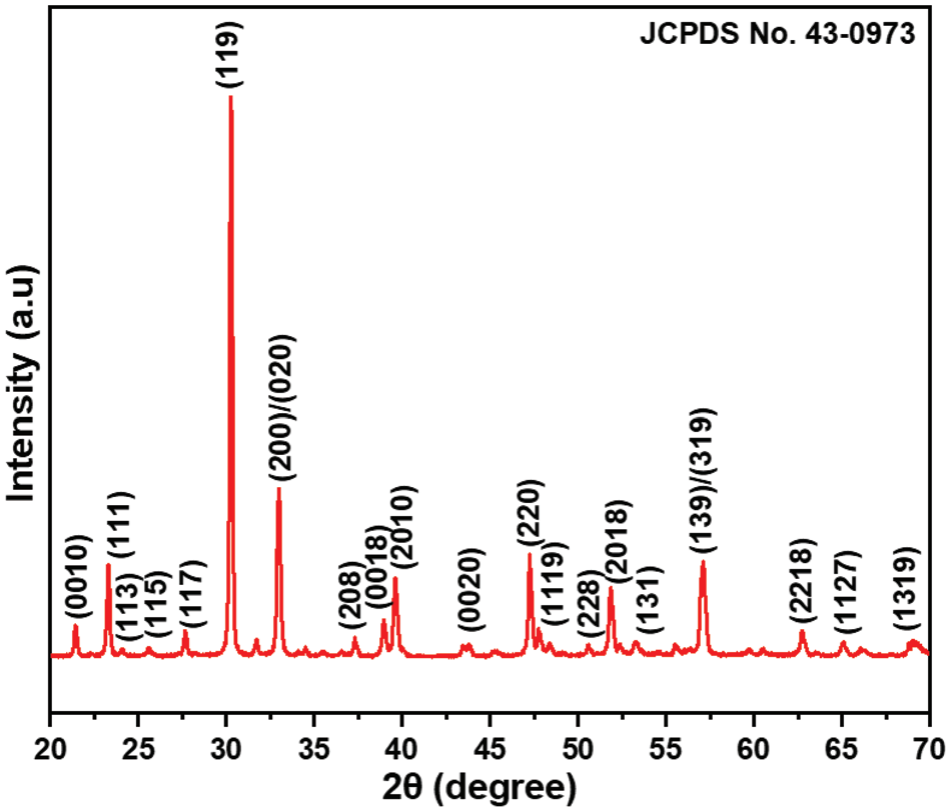
X‐ray diffraction (XRD) plot of BBT.

XRD data of BBT powder were plotted against interference intensity on XRD machine detector versus X‐ray incident angle (2θ) as shown in **Figure** **1**. Peaks were matched with the international center for diffraction data (ICDD). JCPDS number 43–0973 was matched and analyzed. BBT powder showed a single‐phase orthorhombic structure with space group A21am. No impurity is detected in BBT powder indicates formation of BBT powder. Priyambada et al. estimated lattice data by CHEKCELL program and assigned *a* = 5.4378, *b* = 5.4385 and *c* = 41.6264 Å.^[^ ^27^, ^28^ ^]^

**Figure 2:**
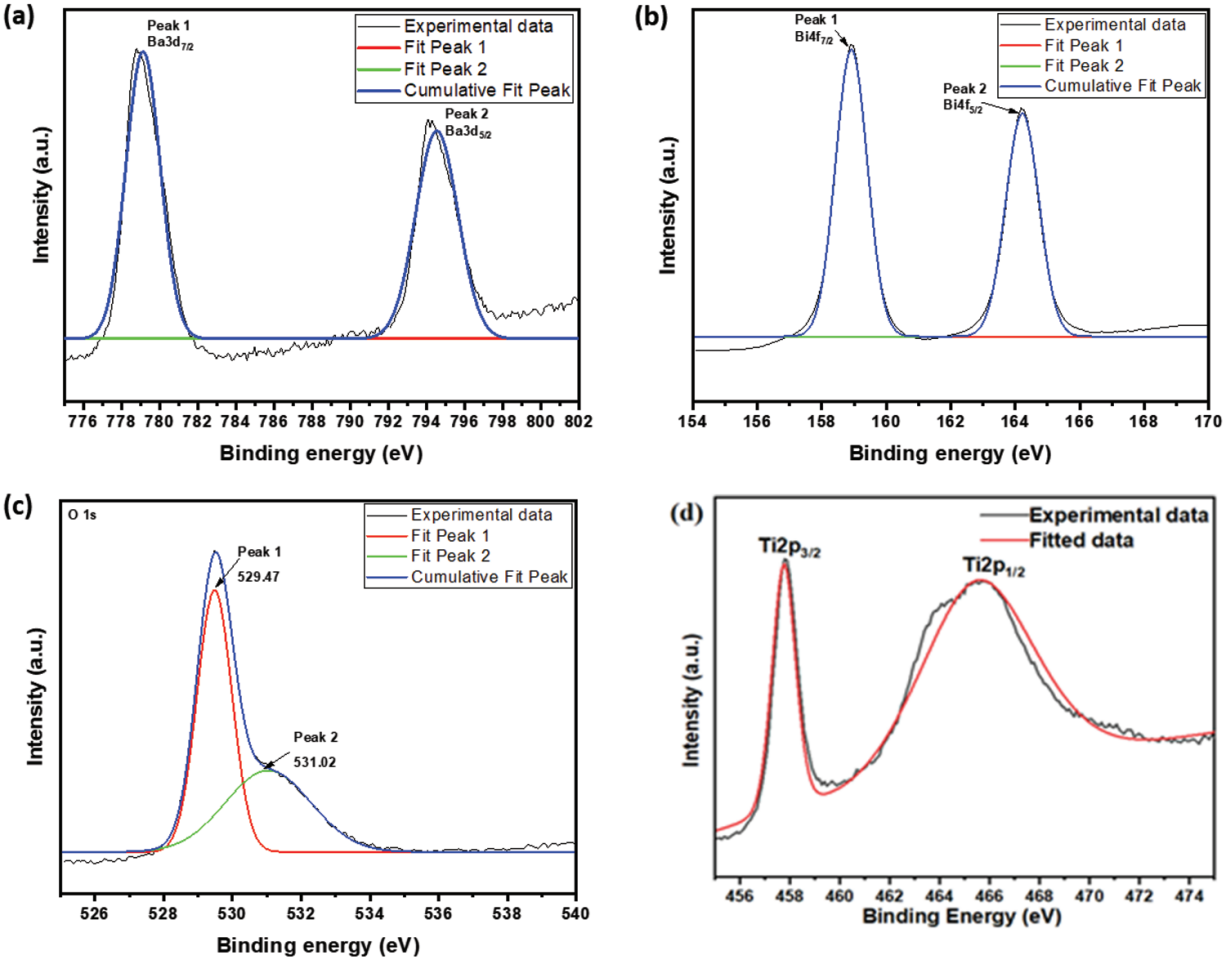
X‐ray photoelectron spectroscopy (XPS) of a) Ba3d, b) Bi4f, c) O1s, and d) Ti2p.

**Figure** **2** elaborates the XPS spectrum of the BBT powder and the XPS chemical states of C, Ba, Bi, Ti, and O elements. Figure 2 reveals that there are four characteristic peaks which are assigned to Ba, Bi, Ti and O elements. Figure 2a shows the spectrum of Ba 3d. Binding energy peaks, that is, 794.28 and 779.2 eV, are assigned to Ba atoms in the BBT perovskite phase. They are assigned to Ba 3d_7/2_ and Ba 3d_5/2_ states with an energy separation about 15.08 eV. The XPS spectrum of Ti 2p (Figure 2d) is deconvoluted into two peaks at the binding energies of 465.68 and 457.78 eV, respectively. They are assigned to Ti 2p_1/2_ and Ti 2p_3/2_ states with an energy separation about 7.9 eV. The O 1 s core‐level spectrum (Figure 2c) can be fitted at 529.7 and 531.02 eV. The peak at 529.7 eV is assigned to the metal—O bond in BBT perovskite structure. Figure 2b shows the spectrum of Bi 4f. Binding energy peaks, that is, 164.18 and 158.88 eV, are assigned to Ba atoms in the BBT perovskite phase. They are assigned to Ba 4f_5/2_ and Ba 4f_7/2_ states with an energy separation about 5.3 eV. In summary, the valence states of Ba, Bi, Ti, and O elements in the powder are in the states of Ba2^+^, Bi2^+^, Ti4^+^, and O2^−^, respectively.

**Figure 3:**
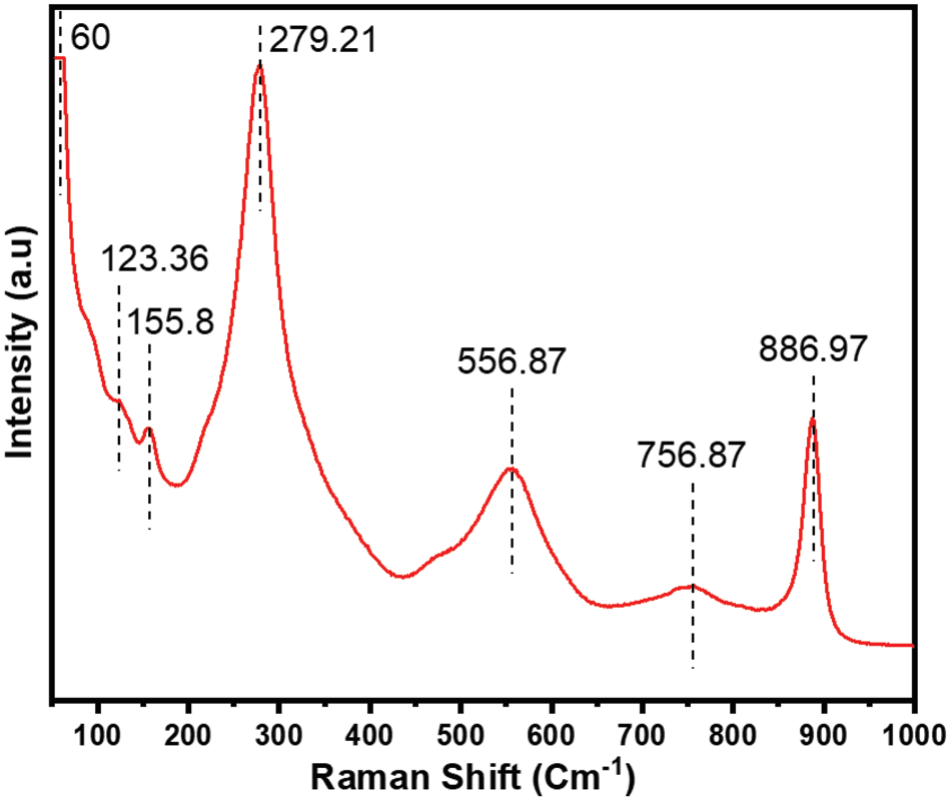
Raman spectrum of BBT.

**Figure** **3** illustrates Raman spectrum of BBT powder. Vibrational modes of BBT were analyzed by observing the presence of Ti—O, Bi—O, and Ba—O. These vibrational modes were characteristics of BBT of orthorhombic structure.^[^ ^29^ ^]^ Vibrational modes located at wave numbers of 279.21, 556.87, and 756.87 cm^−1^ are related to Ti—O bonds. Wave numbers of 60 and 123.36 cm^−1^ are indicating the presence of Bi—O bond. Peak encountered at 886.97 cm^−1^ is indicating the presence of Ba—O bond.

Raman spectrum obtained for BBT powder calcinated at 950 °C was obtained at room temperature. Vibrational modes showed by BBT powder indicate that it has characteristics of an Aurivillius ceramic family with *n* = 4. Ti—O, Bi—O, and Ba—O bonds were indicated by Raman spectrum for synthesized BBT powder which are in good agreement with study performed by Kumar.^[^ ^29^ ^]^

**Figure 4:**
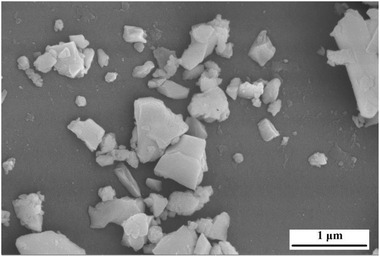
Scanning electron micrograph (SEM) of BBT.

SEM of gold coated and drop casted BBT powder were taken which can be seen in **Figure** **4**. BBT powder particles are brighter in contrast and dark contrast is silicon wafer (background) which was used for drop casting. The particle size of BBT is not the same, it is varying from 100 to 300 nm.

**Figure 5:**
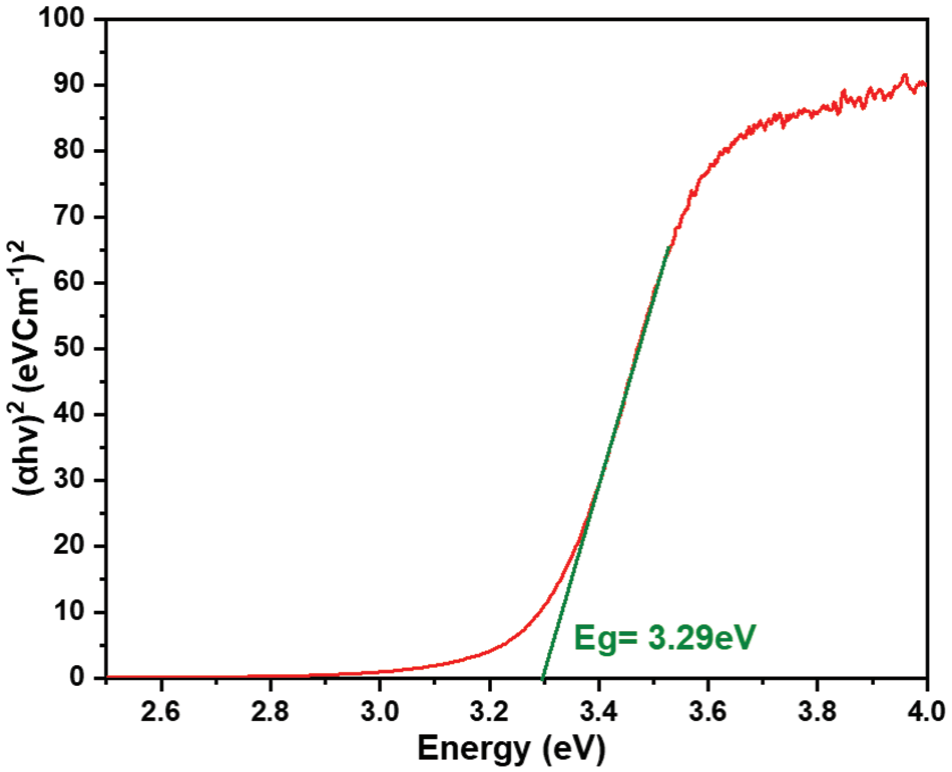
Tauc plot for BBT.

**Figure** **5** shows Tauc's plot for BBT powder. Plot indicated that BBT powder has 3.29 eV of direct optical bandgap. Electron in valence band needs 3.29 eV of energy to jump into conduction band. Jumping of electron from valence band to conduction band makes BBT powder to generate electrons and holes. These electron and holes will be responsible for catalytic behavior of BBT powder. The energy needed for jump of electron is given by in the form of photons or vibrations or both. If energy is given in the form of photon, then that catalytic reaction will be photocatalysis. 3.29 eV of energy comes in the range of ultra‐violet type of radiation. This is the reason that the photocatalysis degradation was performed in the presence of UV light radiation. If energy is given in the form of vibrations that catalytic reaction will be piezocatalysis. 3.29 eV of energy comes in the range of ultrasound type of vibrations. This is the reason that piezocatalysis degradation was performed in the ultrasonicator. Photo–piezo catalysis uses both type of stimuli, that is, UV radiation and ultrasonic vibrations, simultaneously. This is the reason so ultrasonicator was placed under UV radiation while performing photo–piezocatalysis experiments.

### 2.2 Effect of Poling on Catalyst

**Figure 6:**
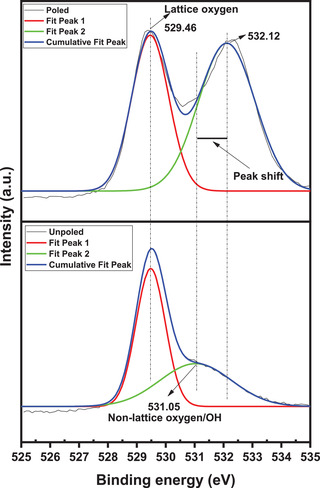
Comparison of XPS spectra of BBT before and after poling.

XPS was recorded after poling and compared with XPS spectrum of unpoled sample. There is a change in spectrum of 1 s oxygen. The XPS spectrum and its comparison with unpoled sample are shown in the **Figure** **6**. As XPS spectrum of unpoled sample shows that there are two peaks, that is, 529.46 and 531.05 eV. These peaks are assigned as lattice oxygen and non‐lattice oxygen respectively (—OH). When XPS spectrum of unpoled BBT is compared with poled BBT it was observed that there is a non‐lattice oxygen peak shift (531.05 to 532.12 eV) of 1.07 eV. The peak is shifting toward higher binding energy. This indicates that the oxidation state of non‐lattice oxygen is increasing. From this observation, it is concluded that there should be formation of new bonds. It was also observed that the area under the curve of non‐lattice oxygen peaks is also increasing when compared with unpoled BBT. By these observations it is concluded that there is formation of vacancies due to poling of BBT sample.^[^ ^30^ ^]^ These vacancies also increase the catalysis process.

### 2.3 Dye Degradation Study

#### 2.3.1 Photocatalysis Study

Photocatalytic performance was evaluated for MB dye aqueous solution in deionized water. Concentration of MB dye in aqueous was 5 mg L^−1^. 0.1 g of poled and unpoled BBT powder was used as photocatalyst in 10 mL of dye solution. UV radiation gave enough energy to the electron for jumping from valence band to conduction band. BBT powder was activated in the presence of UV radiation, and generated electron and holes for photocatalytic reaction. Following is a summary of the photocatalytic mechanism employing BBT as a semiconducting material.^[^ ^31^, ^32^, ^33^ ^]^

Photoexcitation: Beginning of photocatalysis process which gives holes in VB and electron in CB. Therefore, an electron and hole pair (e^−^/h^+^) is produced.

(1) $$\begin{array}{c} \text{BBT}+hv\left(\text{UV}\right)\to \text{BBT}\left(e^{-}\left(\text{CB}\right)+h^{+}\left(\text{VB}\right)\right) \end{array}$$

Water ionization: Water combined with holes to form OH· radical.

(2) $$\begin{array}{c} H_{2}O\left(\text{ads}\right)+h^{+}\left(\text{VB}\right)\to \text{OH}\cdot \left(\text{ads}\right)+h^{+}\left(\text{ads}\right) \end{array}$$

Ionization of oxygen: conversion of oxygen into anionic superoxide radical (O_2_) and keeping the BBT molecule's electrons neutral.

(3) $$\begin{array}{c} O_{2}+e^{-}\left(\text{CB}\right)\to O_{2}^{-\cdot }\left(\text{ads}\right) \end{array}$$

Superoxide's protonation: The surface of the photocatalyst has both oxidation and reduction reactions which occur frequently.

(4) $$\begin{array}{c} O_{2}^{-\cdot }\left(\text{ads}\right)+H^{+}⇆{\text{HOO}}^{\cdot }\left(\text{ads}\right) \end{array}$$

(5) $$\begin{array}{c} 2{\text{HOO}}^{\cdot }\left(\text{ads}\right)\to H_{2}O_{2}\left(\text{ads}\right)+O_{2} \end{array}$$

(6) $$\begin{array}{c} H_{2}O_{2}\left(\text{ads}\right)\to 2{\text{OH}}^{\cdot }\left(\text{ads}\right) \end{array}$$

(7) $$\begin{array}{c} \text{Dye}+{\text{OH}}^{\cdot }\to {\text{CO}}_{2}+H_{2}O\left(\text{dye}\text{​}\;\text{intermediates}\right) \end{array}$$

(8) $$\begin{array}{c} \text{Dye}+h^{+}\left(\text{VB}\right)\to \text{oxidation}\;\text{products} \end{array}$$

(9) $$\begin{array}{c} \text{Dye}+e^{-}\left(\text{CB}\right)\to \text{reduction}\;\text{​}\text{products} \end{array}$$

Random aligned electrical dipoles were aligned with the help of Corona poling. Dipoles were aligned in one direction which results in generation of high surface electric potential, whereas in unpoled samples surface electric potential is low as compared to poled sample. Photocatalytic activity requires interaction of dye molecules with piezoelectric materials. When dye molecule reaches near piezoelectric material, catalytic activity progresses due to surface potential. Poled samples possess high surface electric potential which is responsible high photocatalytic activity in poled samples.^[^ ^34^ ^]^

**Figure 7:**
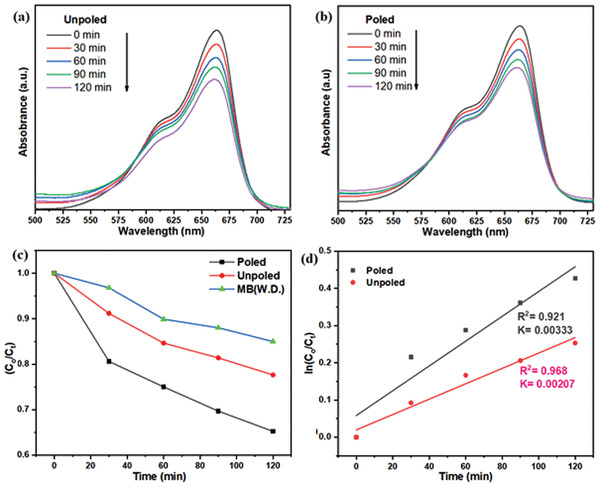
Absorption spectra of 5 mg L^−1^ MB dye (10 mL) photocatalysis with 0.1 g a) unpoled BBT and b) poled BBT. c) C/C_o_ versus time plots and d) ln (C/C_o_) versus time plots for MB dye.

Degradation efficiency was evaluated by observing the absorption spectrum at characteristic wavelength. MB dye characteristic wavelength was observed at 663 nm. **Figure** **7**a,b illustrates absorption spectra of MB dye while performing photocatalysis with unpoled and poled powder of BBT, respectively. Photocatalysis experiment was done for 120 min of UV radiation and absorption spectra were taken after each 30 min of radiation. Absorption value at characteristic wavelength is decreasing after each and every 30 min. This indicates that concentration of MB dye in aqueous solution is continuously decreasing.

Figure 7c illustrates C/C_o_ versus time plots. It can be observed from the plot that poled BBT powder showed higher degradation performance. Degradation of MB dye without catalysis dosage (W.D.) shows self‐degradation of MB dye in the presence of UV radiation. Figure 6d illustrates ln(C/C_o_) versus time of UV radiation. Linear polynomial was fitted to that data point. It indicates that photocatalytic reaction follows the first order of kinetics. Slope of the fitted linear polynomial indicates rate of reaction. It can be observed from Figure 7d that slope is highest for poled powder of BBT. It concludes that the rate of reaction is highest for poled powder of BBT. This is the indication of higher photocatalytic performance of poled powder of BBT.

#### 2.3.2 Piezocatalysis Study

Piezocatalytic performance was evaluated for MB dye aqueous solution in deionized water. Concentration of MB dye in aqueous was 5 mg L^−1^. 0.1 g of poled and unpoled BBT powder was used as piezoocatalyst in 10 mL of dye solution. Piezocatalytic reaction happens when the piezoelectrics stress was exerted under applied deformation caused by the external stress. The polarizing potential with negative and positive charges dispersed on the opposing surfaces of the piezoelectrics will be produced by the nonzero dipole moment established in the crystal lattice with the alteration of the original crystal structure. Numerous cavitation bubbles are produced when an ultrasonic wave is delivered to a liquid continuously, and the quenching of these bubbles causes a significant pressure to be applied to the scattered piezoelectric materials (up to 108 Pa).^[^ ^35^, ^36^ ^]^ The piezo‐induced potential can therefore be produced periodically. It is obvious that rising ultrasonic power encourages catalytic efficiency. Another important element in ultrasonic‐induced piezocatalysis is frequency. The highest rate of energy conversion is achieved at resonance frequency with the maximum vibration amplitude. Ultrasonic vibrations gave enough energy to the electron for jumping from valence band to conduction band. BBT powder was activated in ultrasonic stimuli and, generated electrons and holes for piezocatalytic reaction.

**Figure 8:**
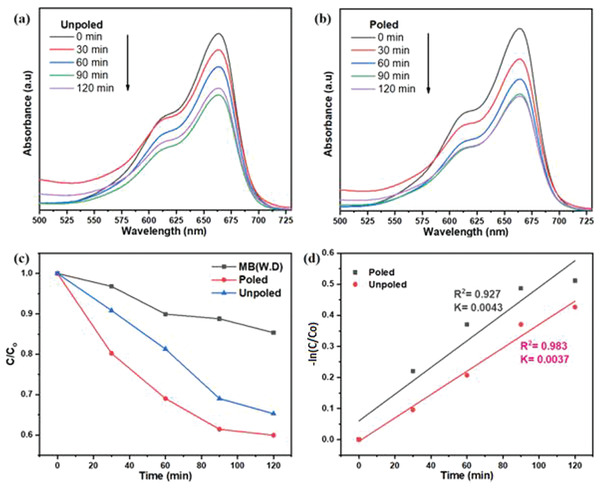
Absorption spectra of 5 mg L^−1^ MB dye (10 mL) piezo catalysis with 0.1 g a) unpoled BBT and b) poled BBT. c) C/C_o_ versus time plots and d) ln (C/C_o_) versus time plots for MB dye.

Degradation efficiency was evaluated by observing absorption spectra at characteristic wavelength. MB dye characteristic wavelength was observed at 663 nm. **Figure** **8**a,b illustrates absorption spectra of MB dye while performing piezocatalysis with unpoled and poled powder of BBT respectively. Piezocatalysis experiment was done for 120 min in the stimuli of ultrasonic vibrations and absorption spectra was taken after each 30 min of ultrasonication. Absorption value at characteristic wavelength is decreasing after each and every 30 min. This indicates that concentration of MB dye in aqueous solution is continuously decreasing.

Figure 8c illustrates C/C_0_ versus time plots under ultrasonication. It can be observed from the plot that poled BBT powder showed higher degradation performance. Degradation of MB dye without catalysis dosage (W.D.) shows self‐degradation of MB dye in the presence of ultrasonic stimuli. Figure 8d illustrates ln (C/C_o_) versus time of ultrasonication. Linear polynomial was fitted to that data point. It indicates that piezocatalytic reaction follows the first order of kinetics. Slope of the fitted linear polynomial indicates rate of reaction. It can be observed from Figure 8d that slope is highest for poled powder of BBT. It concludes that the rate of reaction is highest for poled powder of BBT. This is the indication of higher piezocatalytic performance of poled powder of BBT. It is known that sonocatalysis also triggered dye degradation due to complex phenomena associated with ultrasonication. It is also difficult to quantify contribution from sonocatalysis and intrinsic piezocatalysis. However, as presented in this study, the difference in the performance of poled and unpoled samples is the contribution of materials intrinsic piezoelectric nature.

#### 2.3.3 Photo–Piezo Catalysis Study

Photo–piezo catalytic performance was evaluated for MB dye aqueous solution in deionized water. Concentration of MB dye in aqueous was 5 mg L^−1^. 0.1 g of poled and unpoled BBT powder was used as catalyst in 10 mL of dye solution. Ultrasonic vibrations and UV radiation gave too much energy to the electron for jumping from valence band to conduction band. BBT powder was activated in ultrasonic and UV radiation stimuli and, generated electrons and holes for photo–piezo catalytic reaction. Mechanism of photo–piezo catalysis is combined mechanism of photocatalysis and piezocatalysis as discussed above.

**Figure 9:**
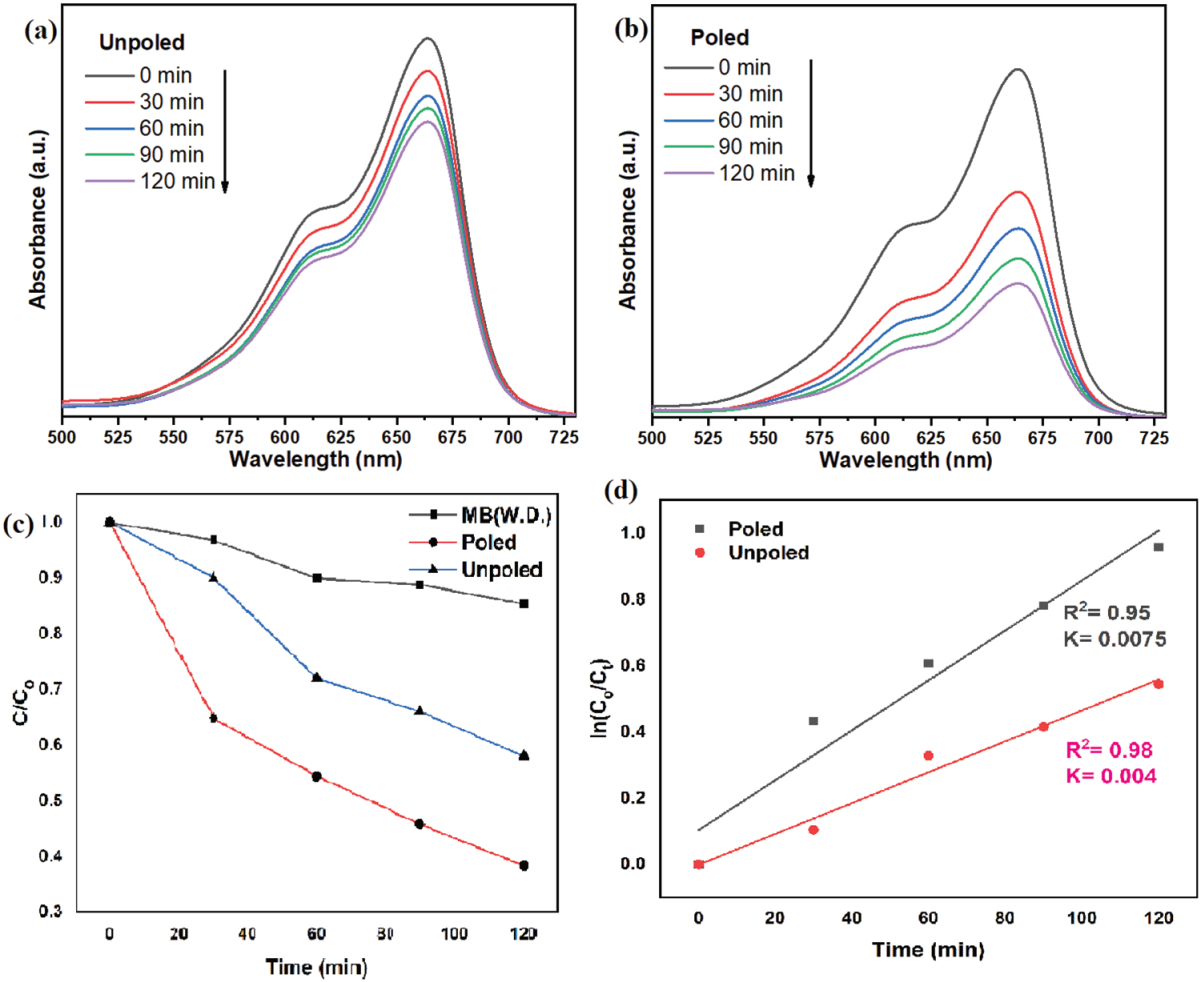
Absorption spectra of 5 mg L^−1^ MB dye (10 mL) photo–piezo catalysis with 0.1 g a) unpoled BBT and b) poled BBT. c) C/C_o_ versus time plots and d) ln (C/C_o_) versus time plots for MB dye.

Degradation efficiency was evaluated by observing absorption spectra at characteristic wavelength. MB dye characteristic wavelength was observed at 663 nm. **Figure** **9**a,b illustrates absorption spectra of MB dye while performing photo–piezo catalysis with unpoled and poled powder of BBT respectively. photo–piezo catalysis experiment was done for 120 min in the stimuli of ultrasonic vibrations along with UV radiation and absorption spectra were taken after each 30 min of reaction time. Absorption value at characteristic wavelength is decreasing after each and every 30 min. This indicates that concentration of MB dye in aqueous solution is continuously decreasing.

Figure 9c illustrates C/C_o_ versus time plots of ultrasonication along with UV radiation plots. It can be observed from the plot that poled BBT powder showed higher degradation performance. Degradation of MB dye without catalysis dosage (W.D.) shows self‐degradation of MB dye in the presence of ultrasonic stimuli along with UV radiation. Figure 8d illustrates ln (C/C_o_) versus time of ultrasonication along with UV radiation. Linear polynomial was fitted to that data point. It indicates that photo–piezo catalytic reaction follows the first order of kinetics. Slope of the fitted linear polynomial indicates rate of reaction. It can be observed from Figure 9d that slope is highest for poled powder of BBT. As it concludes that rate of reaction is highest for poled powder of BBT. This is the indication of higher photo–piezo catalytic performance of poled powder of BBT.

**Figure 10:**
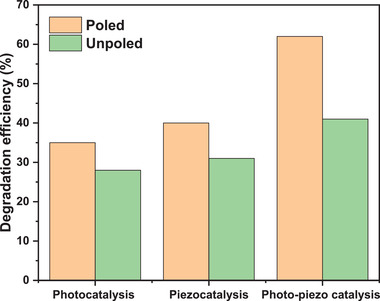
Comparison of degradation efficiencies.

**Figure** **10** illustrates degradation efficiencies of photo, piezo, and photo–piezo catalysis. Photo, piezo, photo–piezo catalysis showed 35% and 28%, 40% and 31%, and 62% and 40% for poled and unpoled BBT powder. Photo–piezo catalysis while using poled BBT powder as catalyst showed highest degradation efficiency. Whereas degradation efficiency of photo–piezo catalysis while using unpoled BBT powder is lowest. This clarifies that there is a significant effect of poling on degradation performance. Two external stimuli were used in photo–piezo catalysis, that is, UV radiation and ultrasonic vibrations which gave ample energy to the electron in the valence band of BBT powder. Electrons take this energy and generate more electron hole pairs compared to single stimuli (UV photons or ultrasonic vibrations). Poled powder of BBT showed highest degradation efficiency because it has permanent electric dipoles in specific directions.^[^ ^34^ ^]^ It develops high surface electric potential on poled BBT catalyst. While unpoled BBT catalyst do not have permanent dipoles arranged in specific directions so it could not generate high surface electric potential on unpoled BBT catalyst.

### 2.4 Scavenger Test

**Figure 11:**
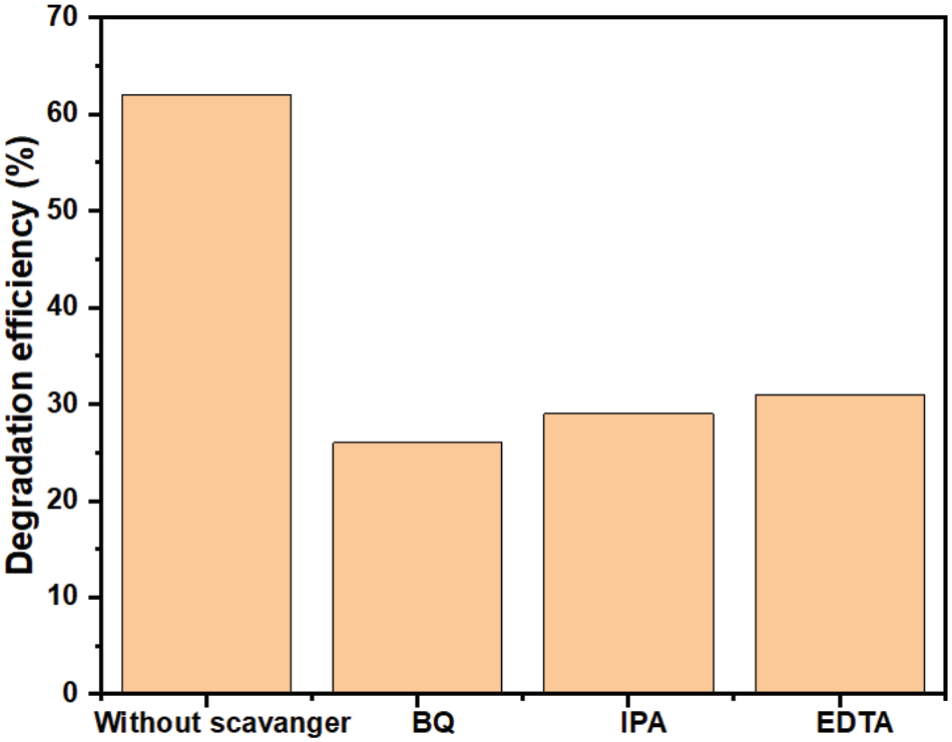
Scavenger test.

Scavenger tests were carried out to identify the major reactive radicals. Same quantity and molarity of scavengers (ethylenediamine [EDTA], isopropyl alcohol [IPA], and benzoquinone [BQ]) were mixed with aqueous MB dye solution. Scavenger tests were performed for best result obtained among photo, piezo, and photo–piezo catalysis. Photo–piezo catalysis showed best results, so scavenger tests were performed in photo–piezo catalysis. EDTA, BQ, and IPA generates h^+^, superoxide radical (.O_2_), and hydroxy radical (.OH).^[^ ^37^, ^38^ ^]^ As shown in **Figure** **11** all three scavengers showed significant decrease in degradation efficiencies as compared to degradation efficiency without scavengers. It indicated that h^+^, .O_2_, and .OH, all three radicals were essential for photo–piezo catalysis. .O_2_ radical is most essential for photo–piezo catalysis than .OH and then h^+^.

### 2.5 Seed germination

**Figure 12:**
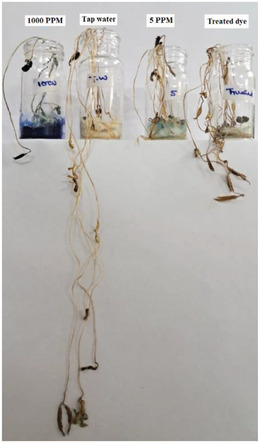
Seed germination using various water (treated and untreated).

Seed germination was also done to give real life evidence of MB dye degradation in aqueous solution. **Figure** **12** illustrates seed germination with the help of tap water, treated MB dye aqueous solution, 5 mg L^−1^ concentration of MB dye in aqueous solution (initial concentration of treated dye solution) and 1000 mg L^−1^ concentration of MB dye in aqueous solution. Different concentrations of MB dye solutions were taken for comparison. Figure 11 indicates that tap water germinated seeds showed highest germination because tap water does not contain any molecule of organic substance (dye) which can lower germination. Treated MB dye aqueous solution showed germinating less than tap water and higher than 5 and 1000 mg L^−1^ MB dye aqueous solution. It is because treated water have MB dye molecule greater than tap water and lower than 5 and 1000 mg L^−1^. 5 mg L^−1^ was the initial concentration of dye which was degraded by photo–piezo catalysis. Treated dye solution grew more seeds as compared to 5 mg L^−1^ dye solution which validates that photo–piezo catalysis is successfully degrading MB dye in aqueous solution.

## 3 Conclusions

BBT was successfully synthesized by using a solid state reaction. BBT powder was characterized by XRD, Raman spectroscopy, XPS, and SEM. Band gap of synthesized material is found to be 3.29 eV by using Tauc's Plot. BBT powder was poled through Corona poling. Photo/piezo/photo–peizo catalytic effect was examined and compared with poled and unpoled samples. 5 ppm MB dye was used for evaluation of catalytic performance. Unpoled samples of BBT showed ≈25%, 28%, and 42% of degradation efficiency with photo/piezo/photo–peizo catalysis respectively. This degradation efficiency was enhanced by poled samples of BBT. Poled samples of BBT showed high degradation efficiency of ≈35%, 40%, and 62% with photo/piezo/photo–peizo catalysis, respectively. Scavenger test showed that h^+^, O_2_, and . OH species are necessary for catalytic reaction. Seed germination showed real life evidence of catalysis. Results show that poling significantly increases catalytic behavior of BBT powder.

## 4 Experimental Section

### Synthesis of BBT Powder

BBT powder was synthesized by solid state reaction. Barium carbonate (BaCO_3_), dibismuth trioxide (Bi_2_O_3_), and titanium dioxide (TiO_2_) were took in stoichiometry ratio and mixed in mortar and pestle with acetone. Mixed powder was calcinated at 950 °C for 4 h.

Crystallographic structure of BBT powder was analyzed by XRD. Rigaku diffractometer was used for the analysis. It used 9 kW Copper K alpha (Cu Kα) anode as target and data of 2θ was recorded in the range of 20° to 70° with rate of 2° min^−1^. Scanning electron micrographs (SEM) were also recorded with the help of FE‐SEM Inspect S50. BBT powder was drop casted on silicon wafer after sonicating it in ethanol. BBT is not a conductive material so gold coating (conductive) was done with the help of megatrone sputtering. Micrographs were taken at 80000× magnification. Chemical structure of BBT powder was analyzed by Raman spectroscopy at room temperature. Raman spectroscopy used for this analysis was of Lab RAM HR evolution model made by Horiba. It used lesser wavelength of 532 nm. Quantitative atomic composition of BBT powder was analyzed by XPS. XPS used for this analysis was +Nexsa base made by Thermofisher Scientific. Optical direct bandgap was evaluated with the help of Tauc's plot. Absorption spectrum of BBT powder was collected with the help of SHIMADZU‐2450, double beam UV–visible spectrophotometer by taking barium sulfate (BaSO_4_) as reference. BBT powder was poled using Corona poling setup (inhouse fabricated). 2 kV mm^−1^ of electric field was applied for 4 h.

### Photo, Piezo, and Photo–Piezo Catalysis Experiments

Dye degradation performance was evaluated with the help of aqueous methyl blue dye. Aqueous solution contained 5 mg L^−1^ concentration of MB dye. 0.1 g BBT poled and unpoled powder were used as catalyst material in 10 mL of MB dye aqueous solution. Dye aqueous solution along with BBT catalyst put in dark for 12 h to achieve equilibrium of absorption for dye molecules on BBT powder surface. Aqueous dye solution was centrifuged at 3000 rpm for 3 min to separate BBT poled/unpoled powder from it. 1 mL of dye solution was carefully taken by pipette for absorption analysis. It was done by SHIMADZU‐2450 UV–visual spectroscopy. Dye solution used for analysis and dye along with BBT powder was mixed with the whole dye solution. Photocatalysis experiments were performed in UV light radiation for 120 min of exposure. 3 UV lamps (Philips 375 nm) of 8 W each were placed 20 cm above the dye solution free surface. Absorption spectra were collected after every 30 min of UV radiation exposure.

Piezocatalysis experiments were also done to evaluate performance. Conventional multipurpose laboratory ultrasonicator of 150 W was used to perform piezocatalysis experiments. Temperature was raised because heat was generated by an ultrasonicator. Temperature was controlled with the help of ice and maintained below 25 °C. Temperature was maintained below 25 °C to restrict pyrocatalysis effect. Piezocatalysis experiments were performed till 120 min. Absorption spectra were recorded after every 30 min of ultrasonication.

Photo–piezo experiments were also done to evaluate performance. In these experiments degradation efficiencies were evaluated under UV radiation and ultrasonic vibrations, simultaneously, so that BBT poled and unpoled powder can be activated because of UV photon and ultrasonic vibrations. 3 UV lamps (Philips 375 nm) were placed above conventional multipurpose laboratory ultrasonicator and experiments were done for 120 min. Temperature was raised because heat was generated by ultrasonicator and UV radiation. Temperature was controlled with the help of ice and maintained below 25 °C. Temperature was maintained below 25 °C to restrict pyrocatalysis effect. Absorption spectra were collected after every 30 min of ultrasonication and UV radiation.

The motivation behind performing piezo and photo–piezo catalysis came from vibration produced by machines inside industries which could be a good source for non‐centrosymmetric material to get polarized which was the basis for piezocatalysis. While for photo–piezo catalysis, solar energy which is a renewable source, when combined with these vibrations, it further enhances dye degrading abilities.

### Scavenger Test

Scavenger test was conducted to evaluate the reactive species in the dye degradation reaction. Test was done for best result of photo/piezo/photo–piezo catalysis with poled and unpoled BBT powder as photocatalyst. Best result of degradation efficiency was obtained for photo–piezo catalysis with poled BBT powder as catalyst. Scavenger test was done with the help of IPA, EDTA, and BQ scavengers for hydroxyl radicals, hole, and superoxide radical, respectively. 0.1 mL of scavengers with 10 mm of molarity was mixed with aqueous of MB dye solution. Photo–piezo catalysis experiments were performed for 120 min of time duration and analyzed by UV–visible spectroscopy.

Absorption spectra were used to evaluate concentration of MB dye in aqueous solution. Concentration of dye in aqueous solution is directly proportional to the numeric value of absorbance at characteristic wavelength. Efficiencies of the degradation were calculated via the following equation.

(10) $$\begin{array}{c} P=\left(C_{o}-C\right)/C_{o}\times 100 \end{array}$$

where *P* is the degradation efficiency (%), *C*_o_ is the initial concentration of dye solution and *C* is the concentration of dye after a specific time period.

### Seed Germination

Seed germination was performed from the best treated MB dye aqueous solution. *Vigna radiata* seed was chosen for germination. Best results were obtained for photo–piezo catalysis with poled BBT powder as catalyst. It was done to show real life evidence of dye degradation. For making it more realistic and comparative, 1000 and 5 mg L^−1^ MB dye aqueous solution and tap water were also used for seed germination. Ten lentil seeds were taken for every dye solution. Seeds with cotton were put in pot and 1 mL of dye solution (treated, 5 mg L^−1^, 1000 mg L^−1^, and tap water) used for watering seeds for everyday till 5 days. After 5 days of watering, seed germination growth was observed.

## Conflict of Interest

The authors declare no conflict of interest.

## Data Availability

The data that support the findings of this study are available from the corresponding author upon reasonable request.
